# Effectiveness of exercise intervention in relieving symptoms of ankylosing spondylitis: A network meta-analysis

**DOI:** 10.1371/journal.pone.0302965

**Published:** 2024-06-14

**Authors:** Yekui Luo, Yonghuan Chen, Xiangning Yan, Lan Zhang, Yuan Shang, Jae Chul Seo

**Affiliations:** 1 Department of Marine Sports, Pukyong National University, Busan, South Korea; 2 Physical Education, Shaanxi College of Communications Technology, Xi’an, China; Uttara Adhunik Medical College, BANGLADESH

## Abstract

**Background:**

Ankylosing spondylitis(AS) is a chronic inflammatory rheumatic disease that leads to a reduced quality of life. Exercise appears to be one of the promising modes of intervention. The aim of this study was to review the available evidence and compare the effectiveness of different exercises in relieving symptoms of AS.

**Methods:**

We searched the Pubmed, WOS, EMbase, CNKI, Cochrane Library, and Scopus databases. The search has spanned from the creation of the database until September 15, 2023. We extracted the first author, year of article publication, sample information, intervention, duration of intervention, and outcome indicators from the literature that met the inclusion criteria. The Cochrane Risk Assessment Tool was used to assess the risk of bias for included studies. I² was used to judge the consistency of the included studies. Egger’s test and Begg’s test were used to judge whether there was significant publication bias. Forest plots were used to compare intervention effects and SUCRA was provided to rank the effects of the interventions. This study has been registered in PROSPERO(No. CRD42024518522).

**Results:**

After screening, 10 papers matched the inclusion criteria for this study, and the total sample size of the 10 papers was 623. Upon analysis, the papers included in this study did not have significant publication bias (Begg’s Test P = 0.210) and had good consistency (P>0.05). The direct comparisons showed that Running, Pilates, Stretching, Yoga, and Tai Chi were more effective interventions than traditional therapies. The effect sizes, confidence intervals, and number of studies for each intervention are shown below: Running [MD -1.90 (95% CI -3.14,-0.66) n = 1], Pilates [MD -1.70 (95% CI -2.90,-0.51) n = 1], Stretching [MD -1.54 (95% CI -2.21,-0.88) n = 4], Yoga [MD -1.24 (95% CI -2.18,-0.30) n = 1], Tai Chi [MD -0.78 (95% CI -1.44,-0.12) n = 2], Exergame[MD -0.80 (95% CI -1.99,0.39) n = 1], Swiss balls[MD -1.07 (95% CI -2.58,0.44) n = 1]. The indirect comparisons showed that the range of effect sizes for each sport intervention intersected the null line. Based on cumulative probability, the order of effectiveness of different exercises in relieving AS symptoms is Running, Pilates, Stretching, Yoga, Tai Chi, Exergame, and Swiss ball.

**Conclusion:**

Running, Pilates, Stretching, Yoga, and Tai Chi provided significant relief from AS symptoms. Exergame and Swiss ball were not statistically significant in relieving AS symptoms. There were no significant differences in the effectiveness of different exercise interventions in relieving AS symptoms. Running may have the most beneficial effect on alleviating AS symptoms.

## Introduction

The sacroiliac joint characteristics of patients with AS undergo significant heterogeneity compared to healthy individuals, and the clinical pathogenesis is characterized by physiologic pain and stiffness in the lumbar, hip, and back [1]. As the disease worsens, the spinal joints of AS patients may be destroyed, and in severe cases, deformities of the spine may occur, thus limiting the patient’s physical activities [2].

There are three main types of treatment for AS: surgical, pharmacologic, and non-pharmacologic. In terms of surgical treatment, some means of surgical treatment has significant overall efficacy, although there is a risk of causing multiple complications [3, 4]. In the aspect of non-pharmacological therapy, non-pharmacological therapy includes acupuncture physiotherapy [5], aquatic therapy [6], education on disease knowledge, and exercise therapy [7]. Experts have reached consensus on the effectiveness of exercise intervention in relieving symptoms of AS, suggesting that appropriate participation in exercise is an effective means of helping to treat AS, and they recommend that healthcare practitioners emphasize the importance of exercise in the management of AS [8]. Some scholars believe that exercise for AS patients should include spinal and peripheral joint mobility exercises, muscle strength training, stretching exercises, aerobic exercises, and breathing exercises [9]. Current research suggests that exercise interventions play a positive role in the rehabilitation of AS patients based on pharmacological treatment [10]. The main goal of exercise therapy is to preserve spinal mobility, reduce the incidence of spinal deformity, and alleviate the patient’s physical pain. Deborah Bond’s research suggested that any form of exercise is more beneficial to health recovery than no exercise at all [11]. Several scholars have demonstrated the positive effects of exercise on AS through experiments, such as Swiss ball [12], Pilates [13], aerobic exercise [14], and water exercise [15]. However, most of the current studies on ankylosing spondylitis are direct comparisons, and there is no way to know which form of exercise is most effective in relieving AS symptoms. Therefore, in this paper, non-exercise therapies in non-pharmacological treatments in ankylosing spondylitis are used as conventional treatments, and network meta-analysis is utilized to compare the intervention effects of exercise interventions with those of conventional therapies. It also indirectly compares which exercise interventions have the greatest positive benefit in relieving AS symptoms. Finally, the intervention effects of individual exercises were ranked in the expectation that they would contribute to the choice of exercise prescription for healthcare practitioners and patients.

## Materials and methods

This study strictly controlled the analysis process based on the Statement for the Evaluation of Meta-analysis (PRISMA) published in 2021 and set the screening criteria for the literature used in the study based on the PICOS principles. This study has been registered in PROSPERO(No. CRD42024518522).

Inclusion criteria: 1. The study population (P) was patients with AS. 2. The intervention (I) comprised various types of exercises aligned with conventional therapy, without restrictions on program, time, intensity, or frequency. The exercises included Stretching, Running, Exergame, Tai Chi, Pilates, Yoga, and Swiss balls; the control measure (C) constituted conventional therapy (medication, acupuncture, physiotherapy, public education, doctor’s consultation) or comparison with these interventions. 3. The outcome index (O) is BASFI (Bath AS Functional Index) [16]. 4. The study design (S) involved Randomized Controlled Trials (RCTs).

Exclusion criteria: 1. Literature with incorrect original data or unobtainable valid data; 2. Literature in which routine interventions included any form of exercise intervention; 3. Literature in which data were reported together with other disorders or which included patients with recurrent AS; 4. Literature in which the full text was not available, conference abstracts, and secondary research type of literature; 5. Identical literature retrieved from different databases.

### Literature retrieval strategy

Searches were performed on CNKI, WOS, Cochrane Library, Embase, Scopus, and Pubmed databases. The search was conducted from the database establishment until September 15, 2023 Searches were conducted using a combination of subject terms and free text words. The search terms included Spondylitis Ankylosing, Ankylosing Spondylitis, Ankylosing Spondylarthritis, AS, Exercise, Physical activity, Sport, and Train. We also searched the GreyNet international, Opengrey, and OpenDoar databases. However, no literature was found that met the inclusion criteria.

### Literature screening and data extraction

Initially, two researchers conducted the literature screening, extracting pertinent information from the articles. Following the screening, they exchanged and verified the extracted data. In cases of disagreement, a discussion and judgment phase involved a third researcher. Once a consensus was reached, the information was included in the study. The extracted information encompassed: 1. First author and publication date; 2. Sample size, age, and gender distribution; 3. Details on exercise interventions, conventional treatment measures, and intervention duration; 4. Outcome indicators and corresponding results assessing the patients’ physical status.

### Statistical analysis

The research utilized Review Manager 5.4 and Stata 17.0 for comprehensive data processing and analysis. Risk assessment of the included literature was conducted through the Cochrane Risk Assessment Tool. Seven biases were assessed: randomization, intervention deviation (allocation and adherence effects), missing outcome data, outcome measurement, selective reporting, and others. If bias ratings indicated low risk, the literature was labeled low risk; "some risk" ratings without reaching "high" indicated "some concerns". An evaluation of "some risk" but not "high risk" suggested a medium risk, while a direct "high risk" evaluation labeled it as high. The combined effect sizes of continuous variables were expressed as Mean Difference (MD). Heterogeneity was tested using the I² test and the results were interpreted using a random effects model. If heterogeneity was large, subgroup analysis was used to explore sources of heterogeneity. Sensitivity analyses were used to assess the stability of the results. The publication bias of the literature was determined by Egger’s test and Begg’s Test, and *P* > 0.05 means that there was no significant publication bias. The network evidence graph illustrates interventions used in the included studies. The graph’s lines and nodes signify distinct interpretations. Line width signifies the number of studies directly comparing interventions, positively correlated with the number of included literature. When conducting net meta-analysis, interventions with closed loops were applied with global inconsistency tests and local inconsistency tests. The results of the direct and indirect comparisons between the interventions are presented in forest plots with a null line of "0" and interpreted based on effect sizes and 95% confidence intervals. The effectiveness ranking of each intervention in relieving AS symptoms relied on SUCRA values and cumulative probability. A larger area under the curve in the cumulative probability plot means better relief for ankylosing spondylitis. The quality of evidence was assessed by using the GRADE tool.

## Results

### Basic features of the included literature

**Fig 1:**
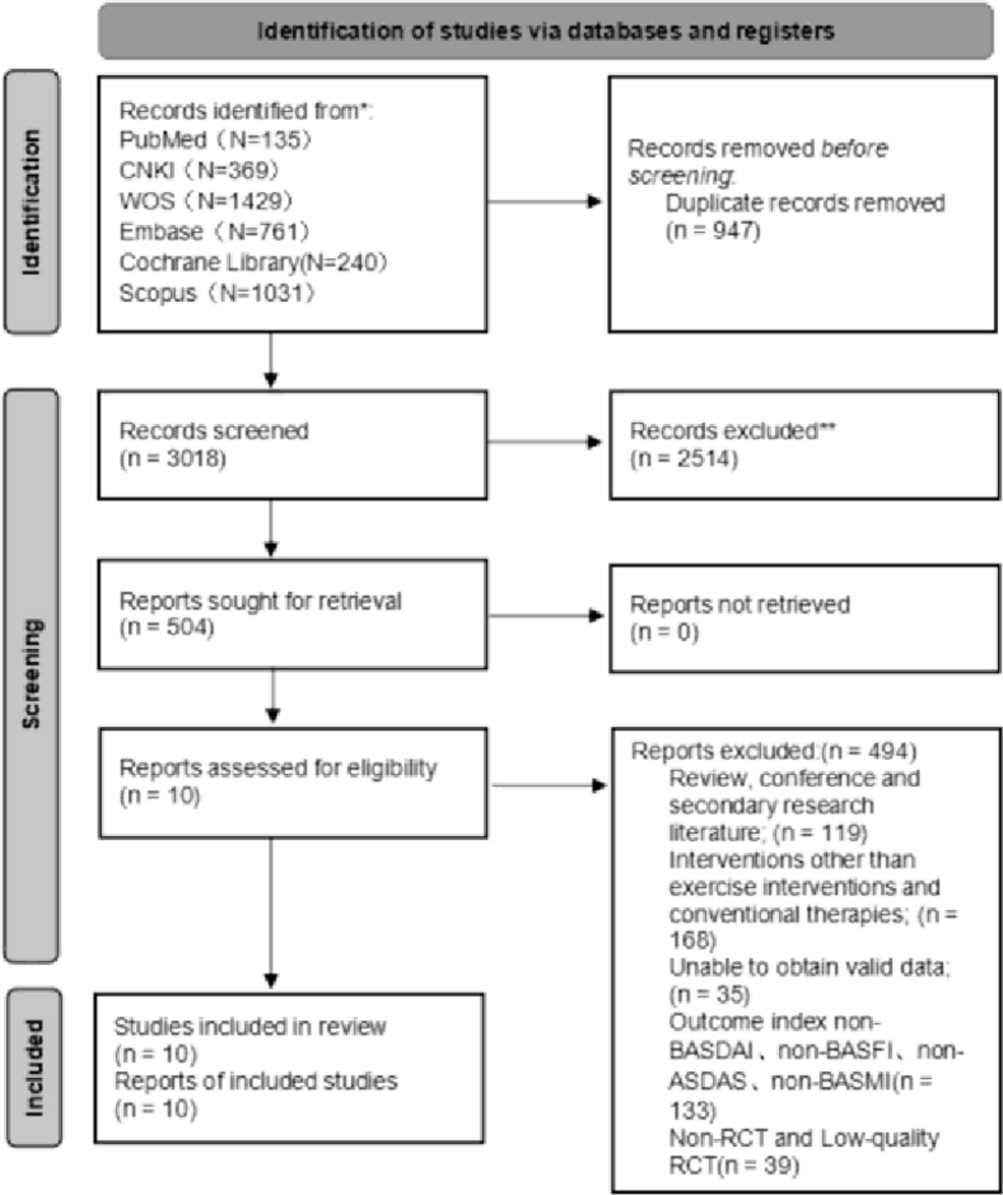
PRISMA 2020 flow diagram.

A search across various databases yielded 3965 relevant research papers. After screening the papers according to the inclusion and exclusion criteria of this study, 10 papers finally met the requirements of this study, as shown in (Fig 1). The experimental group was patients with AS who received the exercise intervention; the control group was patients with AS who received only conventional therapy. The subjects of the study were patients suffering from AS, and the total sample size from the 10 papers was 623, with 327(52.49%) in the experimental group and 296(47.51%) in the control group. The number of males in the sample was 483 (77.53%) and the number of females was 140 (22.47%). The outcome indicator is BASDAI, BASMI, and BASFI. Among these studies, four used Stretch as the intervention; one each used Running, Exergame, Pilates, Yoga, and Swiss balls; and two utilized Tai Chi as the intervention. Farzaneh Gandomi’s study compared Stretch and Pilates interventions with Conventional therapy. Table 1 presents the basic information of the included literature.

**Table 1:** Basic features of the included literature.

First author and year of publication	Sample size T/C	Gender M/F	Age T/C	Intervention T/C	Intervention duration /week	Outcome measures
Rube´n Ferna´ndez Garcı´a (2015) [17]	15/15	16/14	43.8±9.1/ 50±13	Stretch/ Conventional therapy	8	BASDAI、BASFI
Bilge Basakci Calik (2020) [14]	17/14	12/19	46.58±11.94/ 42.85±11.07	Running/ Conventional therapy	12	BASDAI、BASMI、BASFI
Ali Yavuz Karahan (2014) [18]	28/29	45/12	36.1±12.4/ 36.6±11.3	Exergame/ Conventional therapy	8	BASDAI、BASFI
Farzaneh Gandomi (2022) [19]	14/14	28/0	39.21±10.25/ 38.07±8.69	Stretch/ Conventional therapy	6	BASFI
	12/14	26/0	42.58±14.18/ 38.07±8.69	Pilates/ Conventional therapy	6	BASFI
Meliha Kasapoglu Aksoy (2017) [20]	20/21	32/9	37.95±9.84/ 37.47±11.09	Stretch/ Conventional therapy	12	BASDAI、BASFI
Jyoti Singh (2012) [21]	57/52	91/18	34.42±9.39/ 35.09±9.86	Yoga/ Conventional therapy	12	BASDAI、BASFI
Marcelo Cardoso de Souza (2016) [12]	27/28	41/14	45±9.8/ 43.8±10.2	Swiss balls/ Conventional therapy	16	BASDAI、BASMI、BASFI
Li Ruqing (2017) [22]	54/40	67/27	35.7±8.7/ 36.5±9.6	Stretch/ Conventional therapy	12	BASDAI、BASFI
Chen Delin (2014) [23]	43/43	61/25	29.58±6.82/ 30.60±6.54	Tai Chi/ Conventional therapy	8	BASDAI、BASFI
Qu Kun (2020) [24]	40/40	78/2	37.24±14.13/ 40.38±10.67	Tai Chi/ Conventional therapy	32	BASDAI、BASMI、BASFI

### Cochrane risk of bias assessment

**Fig 2:**
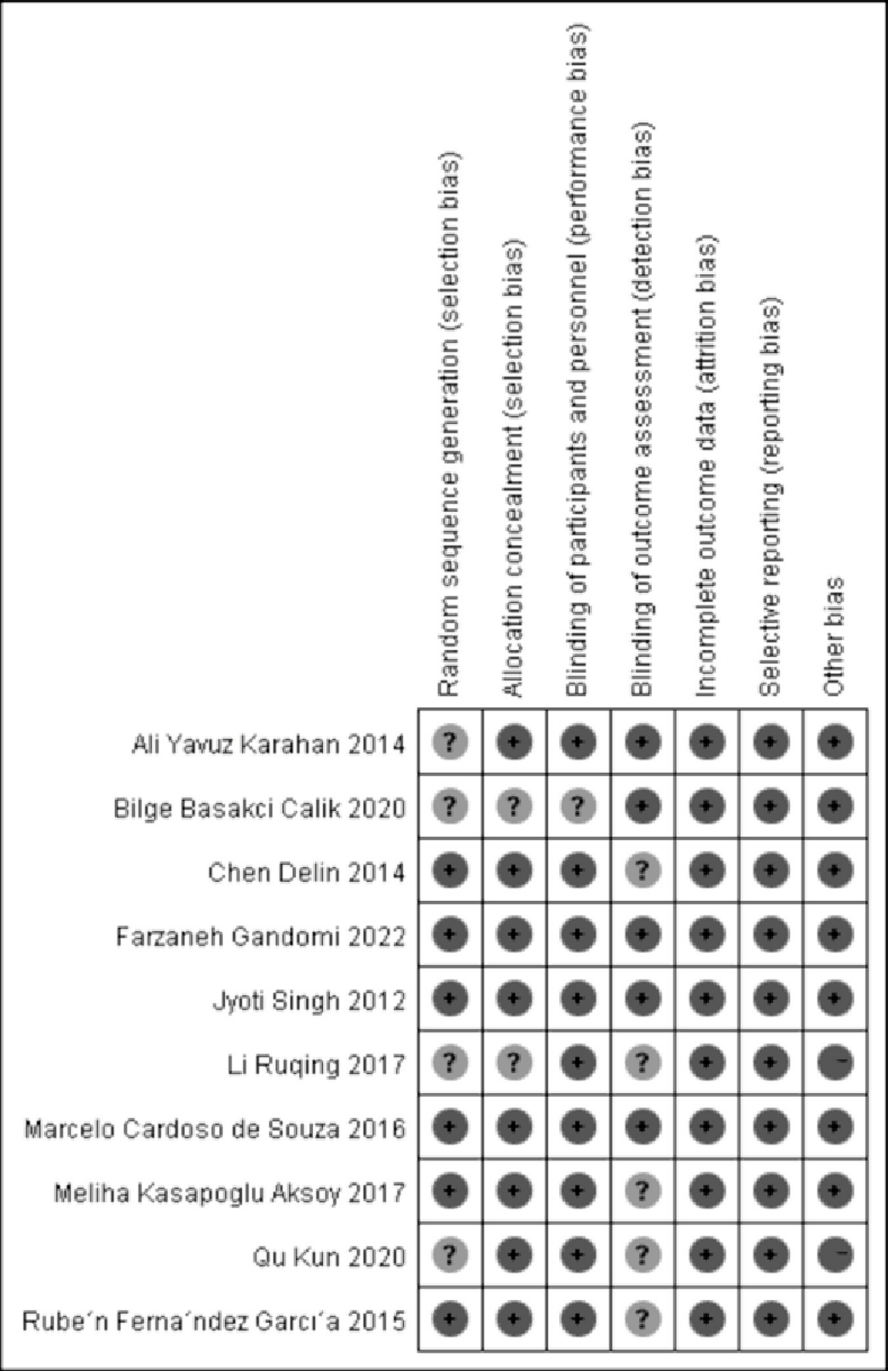
Risk of bias summary.

This study critically evaluated the literature using the Revised Cochrane risk-of-bias tool for randomized trials, RoB2 [25]. (Fig 2) shows the results of the risk assessment of the 10 papers, with "+" indicating low risk, "-" sign indicating high risk, and "?" indicates unknown risk status. The studies by Farzaneh Gandomi, Jyoti Singh, and Marcelo Cardoso de Souza were assessed as low risk, while those by Liu Ruqing and Qu Kun were assessed as high risk. The remaining studies raised some concerns.

### Heterogeneity assessment and publication bias

**Fig 3:**
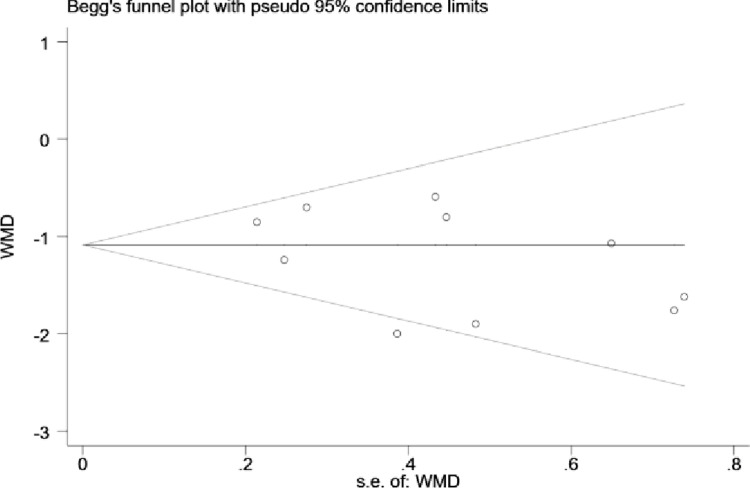
Begg’s test.

The change in data from pre- to post-intervention was computed using data extracted from the 10 eligible papers. Analysis indicated insignificant heterogeneity among the 10 included studies (I² = 40.5%, p = 0.088). In terms of publication bias of the literature, Egger’s test and Begg’s test were applied, and the results of Egger’s test showed p = 0.232, and Begg’s test showed p = 0.210 (Fig 3), indicating that there was no significant publication bias in the included literature. Sensitivity analyses were conducted using leave-one-out approach and the results showed no significant change in any of the significance. Therefore, the results of the study can be judged to be more stable.

### Network evidence map and consistency check

**Fig 4:**
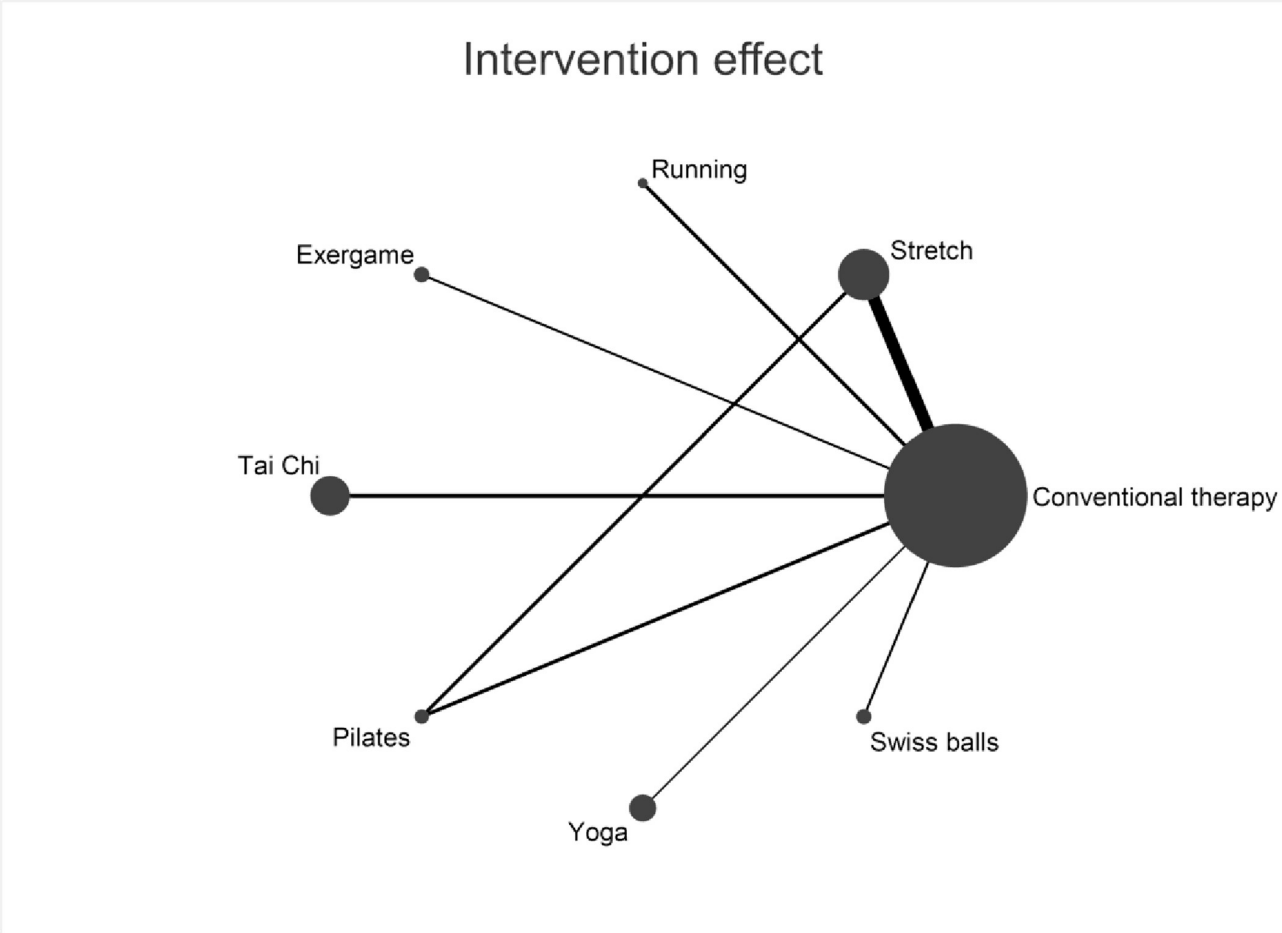
Network evidence for each intervention. Each node represents an intervention. The size of the nodes was positively correlated with the number of times the intervention was used in the experiment. The width of the lines was positively correlated with the number of experiments comparing the two interventions.

In (Fig 4), the widest line between Stretch and Conventional therapy implies the highest number of studies comparing these interventions in the included literature. Larger nodes correspond to larger sample sizes. (Fig 4) depicts a closed loop among Stretch, Pilates, and Conventional therapy. Following this, consistency tests were conducted. Results showed P > 0.05, in global (P = 0.9335), ring (P = 0.897), and local (P = 0.934) inconsistency tests, indicating the absence of inconsistency.

### Relative effect estimation

**Fig 5:**
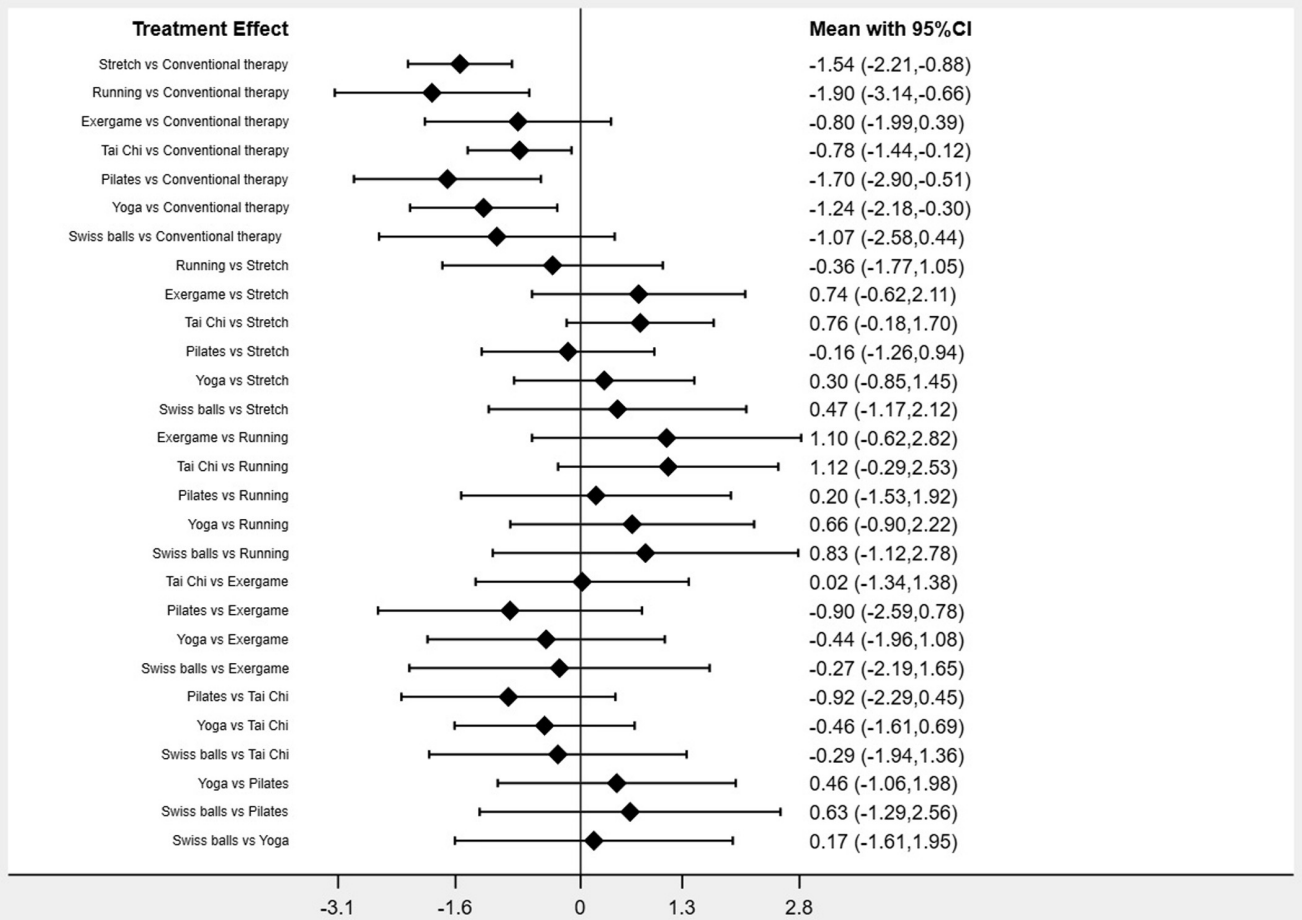
Direct and indirect comparison between interventions.

The results of the direct and indirect comparisons between the various exercise measures at 95%-confidence intervals are shown in (Fig 5). The analysis showed that in direct comparison with conventional therapy, Running [-1.90 (95% CI -3.14,-0.66)], Pilates [-1.70 (95% CI -2.90,-0.51)], Stretch [-1.54 (95% CI -2.21,-0.88)], Yoga [-1.24 (95% CI -2.18,-0.30)] and Tai Chi [-0.78 (95% CI -1.44,-0.12)], implying that these five interventions were better than traditional therapies on alleviating AS symptoms and reached statistically significant differences. However, Exergame [-0.80 (95% CI -1.99,0.39)] and Swiss balls [-1.07 (95% CI -2.58,0.44)], indicated that there is no significant difference in the effectiveness of these two interventions in relieving AS symptoms compared to conventional therapies. Additionally, indirect comparisons revealed no significant differences among individual exercise interventions.

### Ranking probability

**Fig 6:**
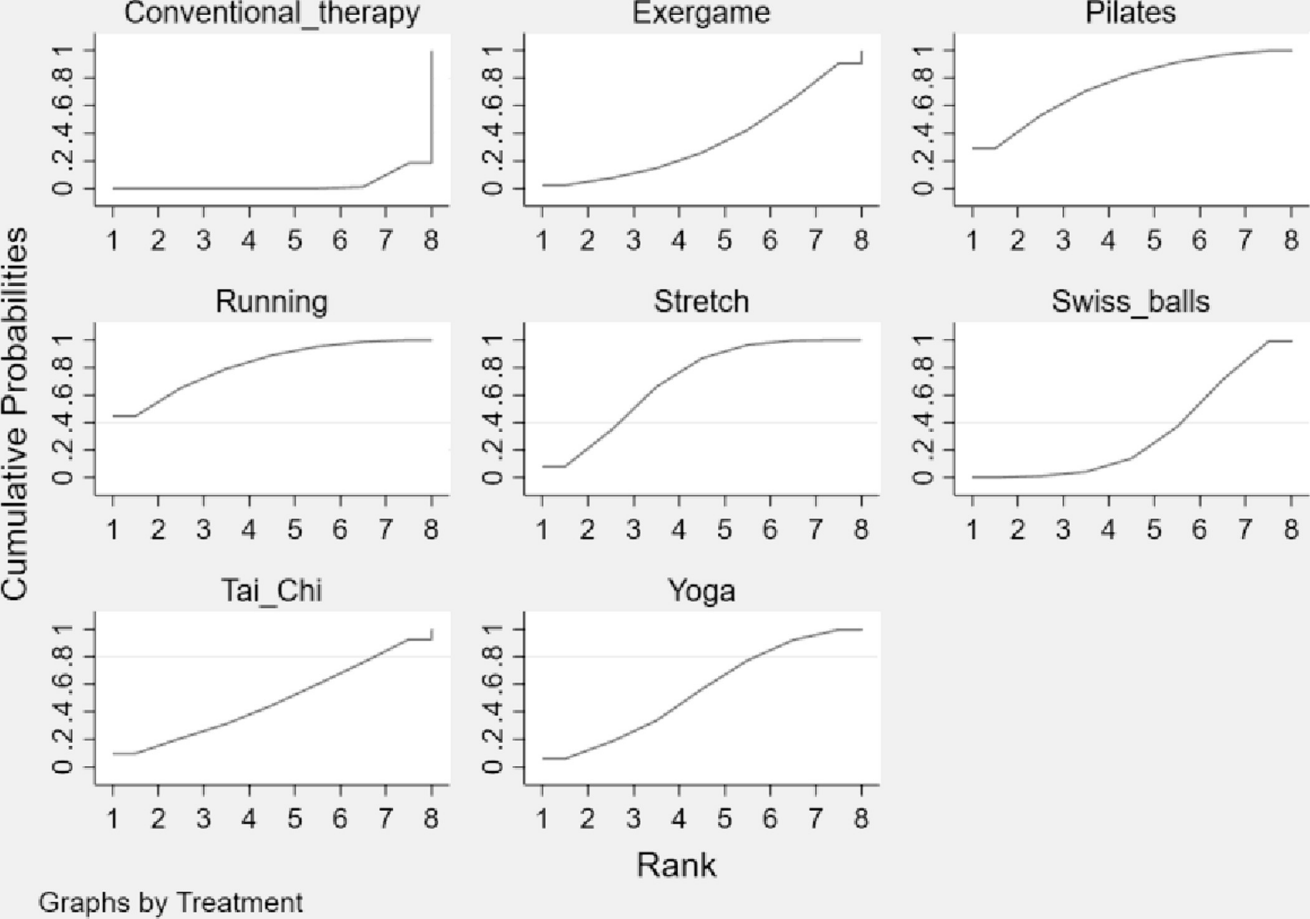
SUCRA curves of the effectiveness of each exercise form. The larger the area under the SUCRA curve, the greater the cumulative probability of ranking high for that intervention.

To know the most effective exercise intervention on alleviating AS symptoms and rank each intervention’s impact, providing guidance for healthcare workers and patients in selecting interventions. In this study, exercise interventions were ranked employing cumulative probability. Relative to the probability of ranking the best, cumulative probability takes into account the uncertainty of relative effect estimation and relative ranking [26], and therefore researchers mostly use this approach for ranking. Table 2 shows the SUCRA value, cumulative probability, and mean rank for each intervention, and the SUCRA value for each intervention corresponds to the area under the curve in the cumulative probability plot. From (Fig 6) and Table 2, it can be learned that the area under the curve is largest for Running (SUCRA = 81.4), followed by Pilates (SUCRA = 75.1), Stretch (SUCRA = 70.1), Yoga (SUCRA = 55.4), Tai Chi (SUCRA = 47.6), Exergame (SUCRA = 35.3) and Swiss balls (SUCRA = 32.4). Thus, it can be judged that Running performed best among all interventions in relieving AS symptoms, followed by Pilates, Stretch, Yoga, Tai Chi, Exergame, Swiss balls, and Conventional therapy in that order.

**Table 2:** SUCRA, PrBest, and MeanRank for each intervention.

Interventions	SUCRA	PrBest/%	MeanRank
Conventional therapy	2.8	0.0	7.8
Stretch	70.1	8.2	3.1
Running	81.4	44.5	2.3
Exergame	35.3	2.5	5.5
Tai Chi	47.6	9.7	4.7
Pilates	75.1	28.9	2.7
Yoga	55.4	6.0	4.1
Swiss balls	32.4	0.1	5.7

### Quality of evidence

The quality of evidence was assessed on the risk of bias, indirectness, inconsistency, imprecision, and publication bias, combined with large effect, dose-response gradient, and plausible confounding. The results in Table 3 show that the quality of evidence for Stretch was assessed as high; the quality of evidence for Running, Pilates, Yoga, and Tai Chi was assessed as moderate; the quality of evidence for Swiss balls was assessed as low; and the quality of evidence for Exergame was assessed as very low.

**Table 3:** GRADE quality of evidence assessment.

Certainty assessment						№ of patients		Effect	Certainty	Importance
Classification of interventions	Risk of bias	Inconsistency	Indirectness	Imprecision	Other considerations	Intervention	Conventional	Absolute (95% CI)		
Running VS Conventional therapy	Serious	not serious	not serious	Serious^2^	strong association	17	14	MD 1.9 lower (2.85 lower to 0.95 lower)	⨁⨁⨁ Moderate	IMPORTANT
Pilates VS Conventional therapy	not serious	not serious	not serious	Serious^2^	none	12	14	MD 1.76 lower (3.18 lower to 0.34 lower)	⨁⨁⨁ Moderate	IMPORTANT
Stretch VS Conventional therapy	Serious^1^	not serious	not serious	not serious	strong association	103	90	MD 1.57 lower (2.4 lower to 0.74 lower)	⨁⨁⨁⨁ High	CRITICAL
Yoga VS Conventional therapy	not serious	not serious	not serious	Serious^2^	none	57	52	MD 1.24 lower (1.73 lower to 0.75 lower)	⨁⨁⨁ Moderate	IMPORTANT
Tai Chi VS Conventional therapy	Serious^1^	not serious	not serious	not serious	none	83	83	MD 0.79 lower (1.12 lower to 0.46 lower)	⨁⨁⨁ Moderate	IMPORTANT
Exergame VS Conventional therapy	Serious^1^	Serious^3^	not serious	Serious^2^	none	28	29	MD 0.8 lower (1.67 lower to 0.07 higher)	⨁ Very Low	NOT IMPORTANT
Swiss balls VS Conventional therapy	not serious	Serious^3^	not serious	Serious^2^	none	27	28	MD 1.07 lower (2.34 lower to 0.2 higher)	⨁⨁ Low	NOT IMPORTANT

**Note:** CI: confidence interval; MD: mean difference

^1^ The included studies were at risk of bias in randomization, allocation concealment, or blinding.

^2^ The sample size of the included studies was small.

^3^ The results of this study are not consistent with the original study.

## Discussion

The analysis revealed that the 10 included papers did not have significant heterogeneity (I² = 40.5%, p = 0.088) and did not show significant publication bias Egger’s test (P = 0.232), Begg’s test (P = 0.210). The results of the consistency test showed that the p-values were all greater than 0.05, indicating that there was no significant inconsistency. A direct comparison of the exercise interventions involved in this study revealed that Running, Pilates, Stretch, Yoga, and Tai Chi were significantly better than traditional therapies in relieving AS symptoms, but Exergame and Swiss balls did not show significant differences compared to traditional therapies. Indirect comparisons showed no significant differences between the exercise interventions in relieving AS symptoms. It can be determined by the cumulative probability that Running may have the most beneficial effect on alleviating AS symptoms, followed by Pilates, Stretch, Yoga, Tai Chi, Exergame, and Swiss balls. Assessments of the quality of evidence included risk of bias, indirectness, inconsistency, imprecision, and publication bias, but also large effects, dose-response gradients, and plausible confounding. Risk of randomization, allocation concealment, and blinding bias were present in some of the included studies. The quality of evidence for Stretch was assessed as high; the quality of evidence for Running, Pilates, Yoga, and Tai Chi was assessed as moderate; the quality of evidence for Swiss balls was assessed as low; and the quality of evidence for Exergame was assessed as very low.

The effect of exercise on human health is obvious, and the lack of exercise will reduce the spinal mobility of AS patients, which will aggravate their condition [27]. Proper exercise rehabilitation helps to relax the muscles, relieve stiffness and spasms, and reduce somatic pain [28]. After analyzing the 10 papers included in this study, it was found that exercise interventions do play a more significant role in promoting recovery in AS patients compared to conventional interventions. This is consistent with the research of other scholars [7, 14, 29–31]. In a survey, the researchers found that 68.6% of people with AS had received advice from their doctors about promoting recovery with exercise; and that people with AS who participated in exercise more often than or equal to three times per week had better recovery effects compared to people with AS who participated less often than three times per week [32]. The results of this study also indicate that running is more effective than other forms of exercise intervention for the rehabilitation of AS patients. Researchers who used running as an intervention in the literature included in this study used 55%-80% aerobic interventions. Therefore aerobic exercise may be more effective in the rehabilitation of AS patients. In 1996, Carbon found that aerobic exercise played a positive role in the recovery of AS patients [33]. In Karin’s experiment, aerobic exercise was found to significantly improve fitness and reduce peripheral pain in AS patients [34]. Some researchers have also demonstrated that aerobic exercise has a significant effect on promoting the recovery of AS patients [35, 36]. Meanwhile, Yoga [37], Pilates [13], and Stretching [38] have become popular rehabilitation tools because of their positive effects on muscular and skeletal-type disorders. During the practice of Tai Chi, deep diaphragmatic breathing is integrated into the body’s movements to achieve a balance between the mind and body [39]. And during the practice of Tai Chi, the intensity of exercise can be adjusted by changing the speed of practice and body posture, thus playing an active role in the prevention and rehabilitation of chronic diseases [40]. However, the results of this study also showed that Exergame and Swiss Ball were not as effective in relieving the symptoms of AS when compared to traditional therapies. Only two studies were found in the searchable literature using these two interventions for people with AS. Therefore, Until more high-quality literature is found on the use of these two types of exercise as interventions, we will not be able to determine the reasons for the emergence of such results. Meanwhile, among the included literature, only the researchers who used running as an intervention reported the intensity of the exercise in their articles (patients continued to exercise at 55–80% of their maximum heart rate for 40 minutes). This intensity seems to be greater than the other exercise interventions, so we can infer that slightly more intense aerobic interventions may be more beneficial for patients with AS. However, this inference cannot be confirmed at this time and therefore needs to be continued to be complemented by future researchers.

There are also limitations in our study. The first is the small number of studies included, with only one paper each using Exergame, Swiss ball, Pilates, Yoga, and Running as interventions. Secondly, in the included studies, the duration and intensity of the researchers’ interventions for patients with AS were different, which may also affect the rehabilitation outcomes of patients with AS, thus leading to biased results in the meta-analysis. In addition, some of the included studies were at risk of bias in terms of the randomization process, allocation concealment, etc., and therefore these studies should be treated with caution. Finally, our study found that Exergame and Swiss Ball did not show significant relief of AS symptoms compared to conventional therapies. This is contrary to the initial findings, but due to the limited number of included studies, we were unable to determine the reasons for this result. Therefore, this result should be interpreted with caution.

## Supporting information

S1 File List of raw analysis data. (DOCX)

S2 File Complete league table. (DOCX)

S3 File SUCRA data. (DOCX)

S4 File Search strategy. (DOCX)

S1 Checklist PRISMA 2020 checklist. (DOCX)
