# Supplementary material for: Effectiveness of exercise intervention in relieving symptoms of ankylosing spondylitis: A network meta-analysis
Source: PLoS One. 2024 Jun 14;19(6):e0302965. doi: 10.1371/journal.pone.0302965 (PMC11178210; doi:10.1371/journal.pone.0302965)
Supplement: S1 File — (DOCX) [file pone.0302965.s001.docx]

**S1 List of raw analysis data.**

| study | n1 | mean1 | sd1 | n2 | mean2 | sd2 |
| --- | --- | --- | --- | --- | --- | --- |
| Rube´n Ferna´ndez Garcı´a2015 | 15 | -1.87 | 1.672 | 15 | 0.44 | 2.2192 |
| Bilge Basakci Calik2020 | 17 | -2.31 | 1.4136 | 14 | -0.41 | 1.27 |
| Ali Yavuz Karahan2014 | 28 | -0.9 | 1.15 | 29 | -0.1 | 2.1 |
| Farzaneh Gandomi2022 | 14 | -1.47 | 1.2065 | 14 | 0.15 | 2.4872 |
| Farzaneh Gandomi2022 | 12 | -1.61 | 1.0148 | 14 | 0.15 | 2.4872 |
| Meliha Kasapoglu Aksoy2017 | 20 | -0.78 | 1.3784 | 21 | -0.19 | 1.3933 |
| Jyoti Singh2012 | 57 | -1.13 | 1.21 | 52 | 0.11 | 1.36 |
| Marcelo Cardoso de Souza2016 | 27 | -1.26 | 1.7772 | 28 | -0.19 | 2.92 |
| Li Ruqing2017 | 54 | -4.4 | 0.96 | 40 | -2.4 | 2.3 |
| Chen Delin2014 | 43 | -3.62 | 1.0116 | 43 | -2.77 | 0.9715 |
| Qu Kun2020 | 40 | -4.09 | 1.4165 | 40 | -3.39 | 1.0091 |
