# Supplementary material for: Effectiveness of exercise intervention in relieving symptoms of ankylosing spondylitis: A network meta-analysis
Source: PLoS One. 2024 Jun 14;19(6):e0302965. doi: 10.1371/journal.pone.0302965 (PMC11178210; doi:10.1371/journal.pone.0302965)
Supplement: S2 File — (DOCX) [file pone.0302965.s002.docx]

**S2 Complete league table.**

| Running | 0.20 (-1.53,1.92) | 0.36 (-1.05,1.77) | 0.66 (-0.90,2.22) | 1.12 (-0.29,2.53) | 1.10 (-0.62,2.82) | 0.83 (-1.12,2.78) | 1.90 (0.66,3.14) |
| --- | --- | --- | --- | --- | --- | --- | --- |
| -0.20 (-1.92,1.53) | Pilates | 0.16 (-0.94,1.26) | 0.46 (-1.06,1.98) | 0.92 (-0.45,2.29) | 0.90 (-0.78,2.59) | 0.63 (-1.29,2.56) | 1.70 (0.51,2.90) |
| -0.36 (-1.77,1.05) | -0.16 (-1.26,0.94) | Stretch | 0.30 (-0.85,1.45) | 0.76 (-0.18,1.70) | 0.74 (-0.62,2.11) | 0.47 (-1.17,2.12) | 1.54 (0.88,2.21) |
| -0.66 (-2.22,0.90) | -0.46 (-1.98,1.06) | -0.30 (-1.45,0.85) | Yoga | 0.46 (-0.69,1.61) | 0.44 (-1.08,1.96) | 0.17 (-1.61,1.95) | 1.24 (0.30,2.18) |
| -1.12 (-2.53,0.29) | -0.92 (-2.29,0.45) | -0.76 (-1.70,0.18) | -0.46 (-1.61,0.69) | Tai Chi | -0.02 (-1.38,1.34) | -0.29 (-1.94,1.36) | 0.78 (0.12,1.44) |
| -1.10 (-2.82,0.62) | -0.90 (-2.59,0.78) | -0.74 (-2.11,0.62) | -0.44 (-1.96,1.08) | 0.02 (-1.34,1.38) | Exergame | -0.27 (-2.19,1.65) | 0.80 (-0.39,1.99) |
| -0.83 (-2.78,1.12) | -0.63 (-2.56,1.29) | -0.47 (-2.12,1.17) | -0.17 (-1.95,1.61) | 0.29 (-1.36,1.94) | 0.27 (-1.65,2.19) | Swiss balls | 1.07 (-0.44,2.58) |
| -1.90 (-3.14,-0.66) | -1.70 (-2.90,-0.51) | -1.54 (-2.21,-0.88) | -1.24 (-2.18,-0.30) | -0.78 (-1.44,-0.12) | -0.80 (-1.99,0.39) | -1.07 (-2.58,0.44) | Conventional therapy |
