# Supplementary material for: Effectiveness of exercise intervention in relieving symptoms of ankylosing spondylitis: A network meta-analysis
Source: PLoS One. 2024 Jun 14;19(6):e0302965. doi: 10.1371/journal.pone.0302965 (PMC11178210; doi:10.1371/journal.pone.0302965)
Supplement: S3 File — (DOCX) [file pone.0302965.s003.docx]

**S3 SUCRA data.**

effect_t1: Conventional therapy; effect_t2: Stretch; effect_t3: Running; effect_t4: Exergame; effect_t5: Swiss balls; effect_t6: Pilates; effect_t7: Yoga; effect_t8: Tai Chi

| rank | effect_t1 | effect_t2 | effect_t3 | effect_t4 | effect_t5 | effect_t6 | effect_t7 | effect_t8 |
| --- | --- | --- | --- | --- | --- | --- | --- | --- |
| 1 | 0 | 0.082 | 0.445 | 0.025 | 0.001 | 0.289 | 0.06 | 0.097 |
| 2 | 0 | 0.336 | 0.648 | 0.073 | 0.011 | 0.536 | 0.187 | 0.208 |
| 3 | 0 | 0.66 | 0.785 | 0.142 | 0.044 | 0.707 | 0.351 | 0.309 |
| 4 | 0 | 0.869 | 0.884 | 0.256 | 0.14 | 0.834 | 0.575 | 0.441 |
| 5 | 0 | 0.961 | 0.948 | 0.424 | 0.366 | 0.92 | 0.782 | 0.598 |
| 6 | 0.01 | 0.994 | 0.982 | 0.65 | 0.711 | 0.972 | 0.924 | 0.756 |
| 7 | 0.187 | 1 | 0.997 | 0.906 | 0.993 | 0.997 | 0.996 | 0.922 |
| 8 | 1 | 1 | 1 | 1 | 1 | 1 | 1 | 1 |
