# Supplementary material for: Effectiveness of exercise intervention in relieving symptoms of ankylosing spondylitis: A network meta-analysis
Source: PLoS One. 2024 Jun 14;19(6):e0302965. doi: 10.1371/journal.pone.0302965 (PMC11178210; doi:10.1371/journal.pone.0302965)
Supplement: S4 File — (DOCX) [file pone.0302965.s004.docx]

**S4 File. Search strategy.**

PubMed search strategy as an example.

#1 ((("Spondylitis, Ankylosing"[Mesh])) OR (Ankylosing spondylitis[Title/Abstract])) OR (Ankylosing Spondylarthritis[Title/Abstract])

#2 (((Sport[Title/Abstract]) OR (Exercise[Title/Abstract])) OR (Train[Title/Abstract])) OR (Physical activity[Title/Abstract])

#3 Aerobic[Title/Abstract] OR Walking[Title/Abstract] OR Deep water running[Title/Abstract] OR Resistance[Title/Abstract] OR Core stability [Title/Abstract] OR Core control[Title/Abstract] OR Core strength[Title/Abstract] OR Sling exercise[Title/Abstract] OR SE[Title/Abstract] OR Stretching[Title/Abstract] OR Pressure biofeedback[Title/Abstract] OR Stability ball[Title/Abstract] OR Proprioceptive neuromuscular facilitation [Title/Abstract] OR PNF[Title/Abstract] OR McKenzie mechanical diagnosis[Title/Abstract] OR Muscle energy technique[Title/Abstract] OR Whole body vibration[Title/Abstract] OR Aquatic[Title/Abstract] OR High intensity interval training[Title/Abstract] OR Breathing exercise[Title/Abstract] OR Virtual reality exercise[Title/Abstract] OR Tai Chi[Title/Abstract] OR Tai Chi chuan[Title/Abstract] OR Taijiquan[Title/Abstract] OR Yoga[Title/Abstract] OR Pilates[Title/Abstract] OR Combined exercise[Title/Abstract] OR Health Qigong[Title/Abstract] OR Yijinjing[Title/Abstract] OR Wuqinxi[Title/Abstract] OR Liuzijue[Title/Abstract] OR Baduanjin[Title/Abstract] OR Eight section brocade exercise[Title/Abstract]

#4 #2 OR #3

#5 Randomized controlled trial OR Controlled clinical trial OR Randomized OR randomization OR RCT OR (double OR treble OR triple AND (mask OR blind))

#6 #1 AND #4 AND #5

((((("Spondylitis, Ankylosing"[Mesh])) OR (Ankylosing spondylitis[Title/Abstract])) OR (Ankylosing Spondylarthritis[Title/Abstract])) AND (((((Sport[Title/Abstract]) OR (Exercise[Title/Abstract])) OR (Train[Title/Abstract])) OR (Physical activity[Title/Abstract])) OR (Aerobic[Title/Abstract] OR Walking[Title/Abstract] OR Deep water running[Title/Abstract] OR Resistance[Title/Abstract] OR Core stability [Title/Abstract] OR Core control[Title/Abstract] OR Core strength[Title/Abstract] OR Sling exercise[Title/Abstract] OR SE[Title/Abstract] OR Stretching[Title/Abstract] OR Pressure biofeedback[Title/Abstract] OR Stability ball[Title/Abstract] OR Proprioceptive neuromuscular facilitation [Title/Abstract] OR PNF[Title/Abstract] OR McKenzie mechanical diagnosis[Title/Abstract] OR Muscle energy technique[Title/Abstract] OR Whole body vibration[Title/Abstract] OR Aquatic[Title/Abstract] OR High intensity interval training[Title/Abstract] OR Breathing exercise[Title/Abstract] OR Virtual reality exercise[Title/Abstract] OR Tai Chi[Title/Abstract] OR Tai Chi chuan[Title/Abstract] OR Taijiquan[Title/Abstract] OR Yoga[Title/Abstract] OR Pilates[Title/Abstract] OR Combined exercise[Title/Abstract] OR Health Qigong[Title/Abstract] OR Yijinjing[Title/Abstract] OR Wuqinxi[Title/Abstract] OR Liuzijue[Title/Abstract] OR Baduanjin[Title/Abstract] OR Eight section brocade exercise[Title/Abstract]))) AND (Randomized controlled trial OR Controlled clinical trial OR Randomized OR randomization OR RCT OR (double OR treble OR triple AND (mask OR blind)))
