# Supplementary material for: Effectiveness of exercise intervention in relieving symptoms of ankylosing spondylitis: A network meta-analysis
Source: PLoS One. 2024 Jun 14;19(6):e0302965. doi: 10.1371/journal.pone.0302965 (PMC11178210; doi:10.1371/journal.pone.0302965)
Supplement: S1 Checklist — (DOCX) [file pone.0302965.s005.docx]

| **Section and Topic** | **Item #** | **Checklist item** | **Location where item is reported** |
| --- | --- | --- | --- |
| **TITLE** | | |  |
| Title | 1 | This report was identified as a meta-analysis. | 1 |
| **ABSTRACT** | | |  |
| Abstract | 2 | The list of abstracts for PRISMA 2020 has been consulted. | 2 |
| **INTRODUCTION** | | |  |
| Rationale | 3 | This is stated in the introduction | 4 |
| Objectives | 4 | This is described in the introduction. | 7 |
| **METHODS** | | |  |
| Eligibility criteria | 5 | This is described in the Materials and methods section. | 7 |
| Information sources | 6 | Searches were performed on CNKI, WOS, Cochrane Library, EMbase, Scopus, and Pubmed databases. The search was conducted from the database establishment until September 15, 2023 Searches were conducted using a combination of subject terms and free text words. | 8 |
| Search strategy | 7 | Searches were performed on CNKI, WOS, Cochrane Library, EMbase, Scopus, and Pubmed databases. The search strategy will be uploaded as an attachment | 8 |
| Selection process | 8 | This is described in the Literature screening and data extraction section. | 8 |
| Data collection process | 9 | This is described in the Literature screening and data extraction section. | 8 |
| Data items | 10a | This is described in the Materials and methods section. | 7 |
|  | 10b | This is listed in Table 1 | 10 |
| Study risk of bias assessment | 11 | This is described in the Cochrane risk of bias assessment section. | 12 |
| Effect measures | 12 | This is described in the Statistical analysis section. | 9 |
| Synthesis methods | 13a | This is described in the Cochrane risk of bias assessment section. | 12 |
|  | 13b | This is described in the Statistical analysis section. | 9 |
|  | 13c | This is described in the Statistical analysis section. | 9 |
|  | 13d | This is described in the Statistical analysis section. | 9 |
|  | 13e | If heterogeneity was large, subgroup analysis was used to explore sources of heterogeneity. | 9 |
|  | 13f | Sensitivity analysis showed that the study results were stable | 13 |
| Reporting bias assessment | 14 | Egger's test and Begg's Test | 13 |
| Certainty assessment | 15 | This is described in the Statistical analysis section. | 9 |
| **RESULTS** | | |  |
| Study selection | 16a | This is shown in Figure 1 | 10 |
|  | 16b | This is shown in Figure 1 | 10 |
| Study characteristics | 17 | This is listed in Table 1 | 10 |
| Risk of bias in studies | 18 | This is listed in Table 1 | 7 |
| Results of individual studies | 19 | This is described in the Statistical analysis section. | 9 |
| Results of syntheses | 20a | This is described in the Heterogeneity assessment and publication bias section. | 13 |
|  | 20b | This is described in the Statistical analysis section. | 9 |
|  | 20c | There was less heterogeneity in the literature included in this study(I²=44.3%, p=0.056). | 13 |
|  | 20d | Heterogeneity of the included literature was low, and sensitivity analyses showed stable results, so they are not reported separately. |  |
| Reporting biases | 21 | This is described in the Cochrane risk of bias assessment section. | 9 |
| Certainty of evidence | 22 | This will be described in the discussion section. | 18 |
| **DISCUSSION** | | |  |
| Discussion | 23a | This is described in the Discussion section. | 18 |
|  | 23b | This is described in the Discussion section. | 21 |
|  | 23c | This is described in the Discussion section. | 21 |
|  | 23d | This is described in the Discussion section. | 21 |
| **OTHER INFORMATION** | | |  |
| Registration and protocol | 24a | The protocol is described in the Methods. Registration does not apply. |  |
|  | 24b | The protocol is described in the Methods. Registration does not apply. |  |
|  | 24c | The protocol is described in the Methods. Registration does not apply. |  |
| Support | 25 | There was no funding support for this research | 22 |
| Competing interests | 26 | No competing interests | 23 |
| Availability of data, code and other materials | 27 | The datasets used and/or analyzed during the current study are available from the corresponding author upon reasonable request. | 22 |

*From:*  Page MJ, McKenzie JE, Bossuyt PM, Boutron I, Hoffmann TC, Mulrow CD, et al. The PRISMA 2020 statement: an updated guideline for reporting systematic reviews. BMJ 2021;372:n71. doi: 10.1136/bmj.n71

For more information, visit: <http://www.prisma-statement.org/>
